# Graphene oxide nanosheets coupled with paper microfluidics for enhanced on-site airborne trace metal detection

**DOI:** 10.1038/s41378-018-0044-z

**Published:** 2019-02-11

**Authors:** Hao Sun, Yuan Jia, Hui Dong, Longxiang Fan

**Affiliations:** 1 School of Mechanical Engineering and Automation, Fuzhou University, 350116 Fuzhou, China; 2 Fujian Provincial Collaborative Innovation Center of High-End Equipment Manufacturing, 350001 Fuzhou, China; 3 School of Mechanical Engineering, Southeast University, 210096 Nanjing, China

**Keywords:** Engineering, Environmental, health and safety issues

## Abstract

Rapid on-site analysis of airborne trace metals has been heavily favored over traditional methodologies because air pollutants can be altered by environmental, behavioral, and social patterns at any given time and location. However, existing portable approaches are either not capable of performing integrated on-site analysis or not yet practically applicable. Exploiting graphene oxide (GO) in enhancing the analytical performance of paper-based colorimetric detection, for the first time, this paper reports the development of a practically useful portable system for accurate, sensitive on-site characterization of trace metals in ambient particulate matter (PM). The system consists of GO-nanosheet-coated paper devices, unmanned aerial vehicle multiaxial sampling, and cellphone-based colorimetric detection. The increased specific surface area and the homogeneity of color distribution from the coating of GO improves the accuracy and sensitivity of the assays. Additionally, by leveraging a Wi-Fi camera, a self-developed app and a sample pretreatment cartridge, metal in PM samples can be readily processed and characterized on-site within 30 min. The effects of chip geometric design, pH, reaction volume, and metal interference on detection results have been studied. The detection limits of the system were calibrated to be 16.6, 5.1, and 9.9 ng for metals Fe, Cu, and Ni, respectively, which are comparable to the detection limits of commercial inductively coupled plasma (ICP) instruments, thus making our portable system practically useful. Finally, the system was used for airborne trace-metal study at 6 locations in Fuzhou City (China), and the results obtained using our system demonstrated good agreement with those obtained by the ICP. The significance of our system in supplementing air pollution study and furthering research on rapid, accurate, on-site air toxicity assessment was demonstrated.

## Airborne trace metal analysis in your palm

Heavy metals in air can now be promptly and accurately quantified on site. Monitoring the amount of trace metals in air is an important component of environment control, as heavy metals such as Fe, Zn, Mn, and Ni are a health concern when present in large concentrations. While accurate equipment is available for measuring trace metal content in air, most of these are not portable, which makes measuring transient conditions difficult. Now, teams led by Hao Sun from Fuzhou University and Yuan Jia from Southeast University, China, report a portable system that makes use of the large surface area of graphene oxide nanosheets for detecting trace metals. These “paper” devices are paired with a highly portable aerial vehicle and a cell phone application developed by the authors, enabling accurate environmental monitoring at specific sites.

## Introduction

Heavy metals, such as Fe, Zn, Mn, and Ni, are essential for normal growth and development of most living organisms because they are the constituents of many enzymes and other functional proteins^1^. However, elevated concentrations of these metallic elements are considered systemic toxicants, which are known to trigger permanent damage to the health of individuals, even at lower levels of exposure^2–4^. Contemporary research has revealed the toxicities of airborne heavy metals^5^. Specifically, long exposure to airborne trace metals can cause a myriad of human health effects ranging from cardiovascular^6^ and pulmonary inflammation^7^ to cancer^8^ and damage of vital organs^9^. Conventional methods of characterizing trace metals often involve the use of highly sophisticated instruments such as those needed for Atomic Absorption Spectroscopy, Inductively Coupled Plasma (ICP), which are accessible only in centralized laboratories. However, it is known that air pollutants can be affected by environmental, behavioral, and social patterns at any given time and location^10^. It is then difficult to assess such pollutants promptly using conventional methods. Therefore, advanced methods for rapid, on-site characterization of trace metals are still needed^11,12^.

Microfluidic paper-based analytical devices (μPADs) are capable of rapid and cost-effective analytical studies^13–16^. μPADs have been used extensively for the detection of hazardous substances such as heavy metals^17,18^, nitrite^19^, pesticides^20^, ammonium^21^, and drugs^22^ in aqueous media as well as gaseous pollutants such as formaldehyde^23^, acid, and alkali samples^24^. For trace metals analysis, various types of paper analytical systems based on colorimetric detection principles have been demonstrated^25,26^. The practical usefulness of these systems has remained limited due to either the lack of on-site analysis methods or the heterogeneity and less-desirable quality of the color distribution in the detection area, resulting in subpar detection sensitivity and accuracy. The latter is caused mainly by unrestricted flow of the solution containing ligands and reagents towards the edge of detection in the paper surface area upon sample addition. One possible way to resolve this is through surface modification. Nanomaterials have gained considerable attention because of their unique properties that can improve the analytical performance of µPADs^27,28^. A preferred material of choice is graphene oxide (GO). As a derivative of graphene, GO has a theoretical surface area of 2630 m^2^ g^−1^, much higher than that of a cellulose paper^29^. The additional surface area creates more colorimetric assay reaction sites, increasing the binding opportunities and thereby improving the detection sensitivity^30,31^. Additionally, heavy metal cations are known to easily cross the electric double layer of GO and bind to receptors functionalized on the GO^32,33^ surface. This potentially reduces the “washing effect” that is common to any analytical system involving µPADs by introducing an electrostatic binding force between the metal ions and their receptor so that assay color distribution on µPADs is more homogeneous.

With this in mind, by integrating GO nanosheets coated on 3D μPADs, unmanned aerial vehicle (UAV) multiaxial sampling, and on-site cellphone colorimetric detection, this work presents a portable system for accurate, sensitive on-site characterization of trace metals. The increased specific surface area and the homogeneity of color distribution from the GO coating improves both the sensitivity and accuracy of the colorimetric assay. Furthermore, the utilization of 3D paper microfluidic structures allows fluid to move freely in the vertical direction, which also helps improve the homogeneous coloration of reaction zones while enabling multiplexed analytical tests and parallel sample distribution. Finally, relying only on a cellphone-controlled Wi-Fi camera, a self-developed APP and a custom-made sample pretreatment cartridge, in-air collected samples can be readily processed on-site within 30 min. The effectiveness of the GO coating was first verified using Raman spectroscopy and a field-emission scanning electron microscope (FESEM). Next, the effects of chip geometric design, pH, reaction volume, and metal interference on detection results were studied. Then, the portable system was calibrated with 3 metals commonly found in air PM: Fe, Cu, and Ni. The detection limits of these metals were found to be 16.6, 5.1, 9.9 ng, which are comparable to the detection limits of a commercial ICP instrument. Finally, the system was used for characterizing on-site trace metals at 6 locations in Fuzhou City (China); the results obtained using our system were in good agreement with those obtained using the ICP, thus verifying that our portable system is practically useful. The significance of our system in supplementing air pollution information gathering and furthering the progress of rapid, accurate, on-site air toxicity assessment research was demonstrated.

## Results

### System design

**Fig. 1:**
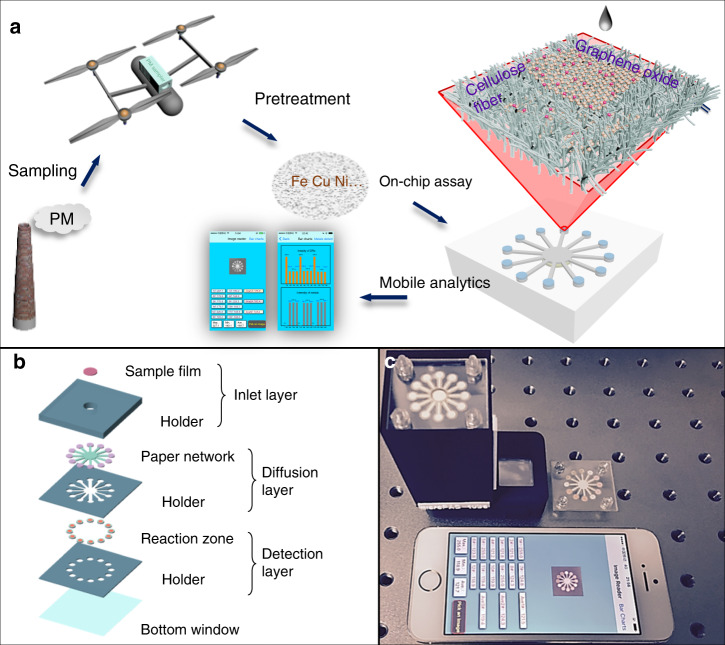
Operation and design of the portable trace-metal detection system. **a** On-site trace-metal quantification operating procedure. **b** Design of the multilayer device. **c** Components of the portable system: assembled 3D microfluidic chip, Wi-Fi camera, hand-held chip bin, and self-developed iOS APP

For the on-site air PM study, the following components were employed: a UAV for on-site airborne PM sampling (Fig. 1a), a 3D printed PM sampler (see Supporting Information, SI, Details of experimental set-up), a 3D chip composed of 3 layers (Fig. 1b), each of which included dedicated paper microfluidic structures that were fabricated using laser-ablated Whatman® filter paper and a corresponding polyethylene terephthalate (PET) holder, a hand-held chip bin (Fig. S2), a cellphone-controlled Wi-Fi camera and an image-processing APP (Fig. 1c). The PET holder also served as an effective seal for the µPADs, exposing only the inlet. This paper also introduces a custom-designed sample pretreatment cartridge (Fig. S3) to facilitate on-site trace metals colorimetric assays. The portable system weighs <1.5 kg, well-suited for easy transportation between testing sites.

### GO deposition and µPAD surface study

**Fig. 2:**
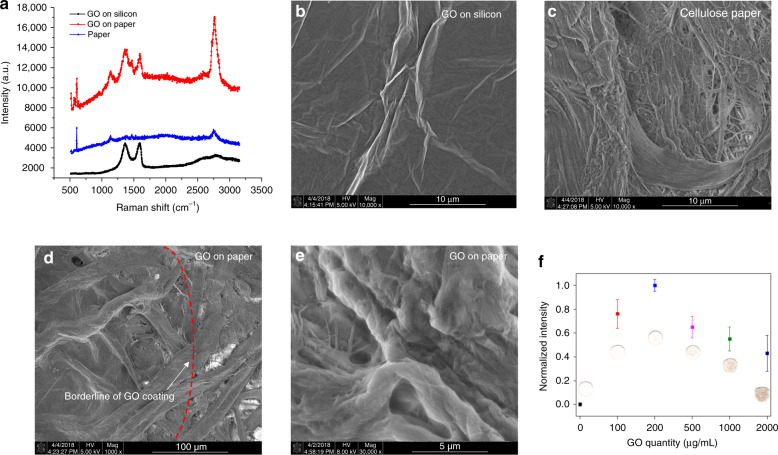
GO characterization. **a** Measured spectra of GO on silicon. **b** Wrinkled GO sheets on silicon. **c** Micrograph of pristine paper. **d** Micrograph of GO on paper with ×1000 and **e** ×30,000 magnification. **f** Intensity of on-chip Fe colorimetric reactions enhanced by different concentrations of GO based on 5 repeated experiments

The first step of the system characterization was to verify the deposition effectiveness of GO. To do so, we employed Raman Spectroscopy and an SEM. First, Raman spectra were acquired of a piece of pristine paper, paper deposited with 200 µg/mL of GO and GO deposited on a silicon substrate (Fig. 2a). Compared with the pristine paper, the Raman spectra of both the GO on paper and GO on silicon showed the G band at 1597 cm^−1^ and the D band at 1365 cm^−1^, which are common for all sp^2^ carbon forms. However, compared with GO on silicon, the Raman spectra of GO on paper additionally displayed a variety of other band peaks that can possibly be attributed to the cellulose paper substrate (the same band peaks were also found on the Raman spectra of pristine paper). Therefore, although characteristic GO bands were found that suggested the successful deposition of GO, due to possible signal interference, the SEM was also used to confirm this.

Corresponding SEM micrographs (Nova NanoSEM 230, Thermo Fisher Scientific) of the abovementioned samples are shown in Fig. 2b–e. Specifically, the GO on silicon sample displays the characteristic GO wrinkle structures as a result of the dried colloidal solution (Fig. 2b), and the micrograph of cellulose paper shows its standard nondirectional fiber structure (Fig. 2c). In addition, to clearly differentiate paper fiber and GO film, the border of the GO colloidal solution was marked during sample preparation and specifically sought during scanning. As a result, Fig. 2d is a good representation of the difference in surface morphology between cellulose fibers with/without a GO coating. Separated by the red dashed line (added in postmicrograph analysis), the region on the left of the micrograph shows a covering film-like feature compared with the region on the right. Additionally, at a higher magnification (Fig. 2e), the feature became more apparent and closely resembles the morphology of GO film deposited on silicon substrates. Therefore, combining the results of Raman spectroscopy and the SEM, the successful deposition of GO onto a paper surface was verified. We also investigated the wettability of GO-modified surfaces (SI: Wettability tests of GO-modified surfaces). It can be concluded that the addition of GO in a concentration below 5000 μg/mL does not affect the wettability of the filter paper significantly and is most likely to make the paper more hydrophilic.

Finally, an optimization process was performed to select the most important condition for producing higher color intensity in the metal assays: the GO colloidal solution concentration. In practice, higher concentrations of GO can potentially induce a strong background color intensity that hinders the detection limit of the assay. For the GO concentration study, different 10 µL concentrations of GO colloidal solutions (100–2000 µg/mL) were prepared and disposed onto the selected paper substrate. Fe metal assays with known concentrations were performed, and the corresponding color intensities were measured. In all cases, the analytical signal was determined by subtracting the background color intensity (Fig. 2f). Although papers soaked with GO colloidal solutions have exhibited increased color signal and uniformity, the optimal concentration with the highest color intensity difference was determined to be 200 µg/mL. This result was expected, as a higher GO concentration is also associated with a strong background color intensity interference, which produced a less favorable detection limit.

### Optimization of experimental parameters

Prior to performing the on-chip tests, the diameter of the detection reservoir and the volumes of the analytes were optimized as follows.

#### Diameter of detection reservoirs

**Fig. 3:**
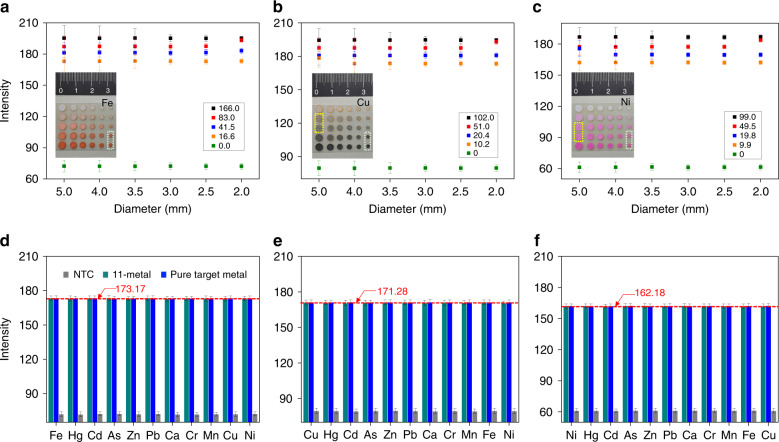
Experimental parameters optimization and metal interference testing. Optimizations of µPADs reservoir sizes using Fe (**a**), Cu (**b**), and Ni (**c**). The results of the interference testing for Fe (**d**), Cu (**e**), and Ni (**f**); error bars were obtained from 5 independent tests. Dashed line indicates the average of pure target metal testing results

The optimization study started by introducing specific volume amounts of well-dispersed GO solution (200 µg/mL) onto paper disks (Whatman® filter paper) with diameters ranging from 2.0 to 5.0 mm and followed by chip drying. Under an optimized pH (SI: pH effect study), specific volume amounts of ligand solutions (in the same concentration) as well as metal solutions (in 4 different concentrations within our LDR, plus a control) were then pipetted sequentially onto the disks to complete a 5 × 6 disk array (Fig. 3a–c). As a result, between the diameters of 2.0 and 5.0 mm, the absolute intensity values for all metals in general did not depend on the disk diameters. However, for diameters of 2.0 mm and smaller, at higher metals concentrations, the corresponding intensities were indistinguishable. In addition, for diameters of 3.5 mm and larger, the standard deviations of the intensities for each concentration increased, which indicated a less-uniform color distribution. Based on the above findings, our selection of a 3.0 mm reservoir diameter is then validated.

#### Analyte volume

The volumes of the analytes, including the volumes of the chelating reagents, and the volumes of deposited GO solutions have been studied for optimizing the performance of the assay. The detailed experimental process is included in SI (chelating reagent volume optimization and GO volume optimization). Briefly, different volumes (0.2–0.6 µL) of a ligand mixture including dithiooxamide (30 mM), sodium acetate buffer (pH 4.0, 20 mM), and 1%(w/w) hydroxylamine in isopropanol were first introduced onto the device, sequentially. Then, using identical amounts of Cu (205 ng), the resulting colorimetric intensity values were obtained and normalized by subtracting the NTC. The optimal volume of ligand mixture was found to be 0.4 µL (Fig. S9a) and was adopted in the following tests. Finally, based on the selected GO concentration (200 μg/mL) above, we studied the optimal volume for GO deposition. Using a similar process, by comparing the intensity values with different GO volumes introduced (0.1–0.6 µL), 0.3 µL was selected for having the highest intensity with the lowest intensity standard deviation (Fig. S9b).

### Metal interference study

The effects of masking reagents for metals ions Fe, Cu, and Ni have been studied recently^34^. The principle of metal interference masking is illustrated in SI (Principle of metal inference masking). To reconfirm, Fe, Cu, and Ni were cross-examined with 8 other commonly found airborne trace metals (i.e., Hg, Cd, As, Zn, Pb, Ca, Cr, and Mn). The masking reagent preparation procedure is illustrated in SI (Metal interference study). The interference study of each target metal started with introducing GO and ligand solution to all 12 DRs of a µPAD in separate steps. Then, metal-specific combinations of masking reagents were introduced to the inlet of the µPAD followed by the addition of the target metal solution. Finally, interfering metals with 10-fold concentration increases and extra-assay controls were introduced to dedicated reservoirs (Fig. S14a, b). Interference testing data for Fe, Cu, and Ni are shown in Fig. 3 d–f, respectively. The tolerance limit^17^ of Fe, Cu, and Ni in the presented experimental setup were all beyond 10:1. In addition, without the intended target metal ion present, there were no apparent colors forming in the detection reservoirs of the µPads, indicating a lack of ligand-metal reactions in NTC samples. Based on these findings, we confirmed the effectiveness of our reagent masking schemes in eliminating possible interference from metal ions in our portable detection system.

### GO-enhanced colorimetric assays

**Fig. 4:**
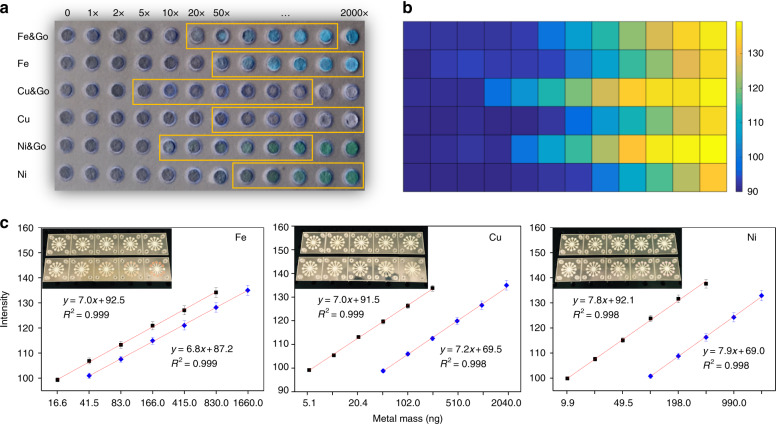
Validation and calibration of GO enhanced on-chip assay. **a** Evaluation of GO enhanced colorimetric assays of Fe, Cu, and Ni. **b** Color representation of the detected assay intensities. **c** On-chip calibration test of three selected metals. Error bar plots were obtained from 5 repeated experiments

Next, we verified our hypothesis that the addition of GO improves the sensitivity of the portable system. To do so, a 6 by 12 array of circular paper microfluidic reservoirs (diameter 3.0 mm) in a PET frame was first fabricated. Of these, the reservoirs in the odd rows were first deposited with 0.3 µL of 200 µg/mL GO colloidal solution and followed with the addition of corresponding metal ligand solutions (Fig. 3a). Ligand-only solutions were introduced in the reservoirs in even rows. In parallel, standard metal-salt solutions were obtained by dissolving 2.0 g of CuSO_4_·5H_2_O, FeCl_3_·6H_2_O, and NiCl_2_·6H_2_O each in 100 mL of DI water. All metal solutions were diluted according to the predetermined concentration gradient (1:2 to 1:2000), and the final concentrations of Cu, Fe, and Ni were determined to be 2.1 ng –4.1 µg, 2.6 ng–5.1 µg, and 2.5 ng–5.0 µg/µL, respectively. Colorimetric assays were then performed by introducing the prepared metal solutions to the reservoir array in order. The results of the assays are shown in Fig. 4a. ImageJ (NIH, Bethesda, MD) was used to analyze the average gray-intensity values of these reservoirs, and the obtained values were plotted using MATLAB (MathWorks, Natick, MA, U.S.) in a colored chart tablet (Fig. 4b). Each block in the chart represents the average gray intensity of each reservoir, and the results were as expected because the intensity values increase monotonically with respect to metal concentrations. More importantly, reservoirs deposited with GO showed improved limits of detection (LOD) for all metals tested (Fe, Cu, and Ni). Of these, the LOD for Fe was improved by a factor of ~2.5, and the LODs for Cu and Ni improved by a factor of 10, thus demonstrating the effectiveness of GO in enhancing the analytical performance of µPADs. Additionally, the linear detection ranges (LDRs), defined as the ranges in which color intensity is directly proportional to metal concentration (highlighted on Fig. 4a) were determined to be 16.6–828.5, 5.1–205.1, and 9.9–496.4 ng for the metals Fe, Cu, and Ni, respectively.

### On-chip metal assay calibration

A set of calibration experiments was performed for the portable system using metal-salt solutions to correlate between the calculated assay gray intensities and the quantity of metal. The detailed protocol of the calibration test is demonstrated in SI (Calibration using metal salt solutions). Metal solutions with concentration gradients identical to the highlighted zones in Fig. 4a were used. Following the experimental procedure described above, an interassay control experiment was first performed with a blank chip deposited with GO and respective ligands to record the background intensity. Next, metal assays were performed one at a time; 10 concentrations were used to complete the calibration for each metal. The calibration experiments began by pipetting 5.0 µL of metal solution with a known concentration onto the sample inlet of a chip. Since there were 12 reaction reservoirs (including an extra negative control reservoir) on a chip, ~0.4 µL of equivalent metal sample was then found in a reservoir, and the amount of metal in the reservoir could thus be calculated accordingly.

After inter/extra background subtraction, the detected average intensities of reservoirs were plotted against metal quantity (Fig. 4c). The corresponding LODs were found to be 16.6, 5.1, and 9.9 ng for the metals Fe, Cu, and Ni, respectively. As a comparison, for µPADs without the GO coating, the metals’ LODs were determined to be to be 41.5, 51.0, and 99.0 ng, respectively, further demonstrating the effectiveness of the GO coating. In addition, the method showed excellent linearity in the selected LDR, which also validated the results from the previous experiments. Once the amount of metal exceeded the upper limit of the LDR, the gray intensity would no longer correlate with metal concentrations. If the amount of metal fell below the lower limit, grayscale intensities obtained from DRs could not be distinguished from the inter/extra assay controls (not shown). Generally, the LDR and LOD of the systems depend on many factors such as the size of the DRs and chip fabrication. For our system, we used a design that attempted to quantify metals in particulate matter in amounts ranging from the nanogram level to the submicrogram level, which is a range generally found in air PM. Furthermore, after multiple tests, the standard deviations of the calibration experiment were found to be 0.13, 0.03, and 0.06 ng, indicating excellent repeatability of the sensing system.

### On-chip multiplex metal assay

**Fig. 5:**
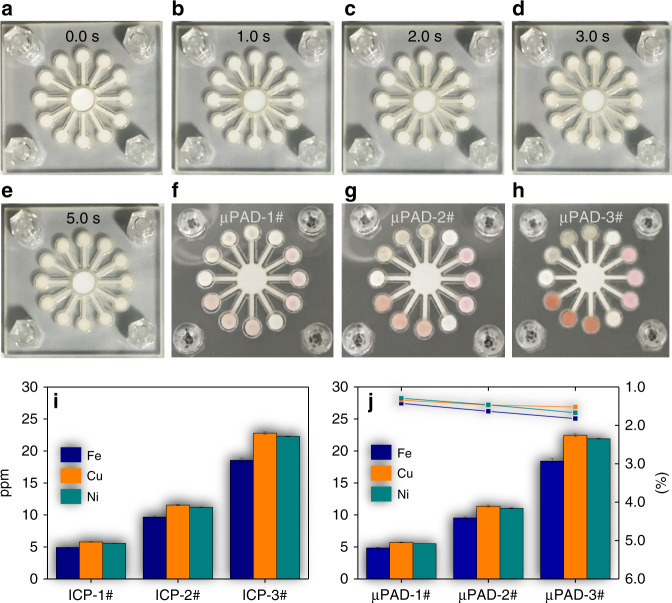
Demonstration of on-chip multiplex metal assays. **a**–**e** Time response of the assay color change in the reaction reservoirs: **a** 0 s, **b** 1 s, **c** 2 s, **d** 3 s, **e** 5 s. **f**–**h** On-chip multiplex assay using metal mixture samples 1–3#. **i** Quantification of metals concentrations of 3 unknown metal solution mixture samples using the ICP. **j** Quantification of identical metal solution samples using the portable system. Error bar plots extracted from 5 different tests

Next, the portable system was calibrated by multiplex metal assays. After preparing the chip using the procedure described above, 5 µL of metal-salt solution mixture that included Cu, Fe, and Ni ions with unknown concentrations were disposed at the inlet of the chip. Masking reagents were used to minimize the interference effect during the multiplex metal assay. The detailed protocol of the multiplex metal test is demonstrated in SI (On-site trace metals multiplex quantification). The time response of the color change as a result of the assays is shown in Fig. 5a–e. In the figure, the assay-induced color changes occurred simultaneously, demonstrating an identical reagent flowrate from the inlet to all DRs. The assay color-intensity change as a result of the metal mixture concentration change is shown in Fig. 5f–h. The portable system was first used to quantify metal concentrations in the mixture solution. In parallel, identical solution samples were sent to a testing facility and quantified by an iCAP™ 7400 ICP-OES Analyzer system (Thermo Fisher Scientific). The quantification results of both methods were plotted in Fig. 5I, j. The on-chip tests and instrument-based tests were each repeated 5 times. The concentrations of Fe in three samples were determined to be 4.91, 9.67, and 18.55 ppm (mass of metal/mass of water) by ICP, and 4.84, 9.54, and 18.42 ppm by our portable system. The corresponding concentrations of Cu were quantified to be 5.78, 11.55, and 22.80 ppm by ICP, and 5.70, 11.38, and 22.45 ppm by our system. Finally, the concentrations of Ni were characterized to be 5.57, 11.20, and 22.28 ppm by ICP, and 5.51, 11.04, and 21.91 ppm by our system. The maximum percent difference of all metal concentrations determined using both methods were calculated to be 1.82, 1.52, and 1.67% for Fe, Cu, and Ni (line plots in Fig. 5j), respectively. In addition, the Student *t*-test and F-test were employed for evaluating the statistical variances of the means of two independent data sets from the on-chip approach and ICP. For Fe, Cu, and Ni, the *P* values were found to be 0.294, 0.575, and 0.485, respectively, which were all above the threshold chosen (0.05 level) for statistical significance, indicating no significant variances of means by the two groups of tests. Detailed testing results and the procedure are demonstrated in SI (Statistical comparison of on-chip and ICP testing results). Therefore, the portable system was calibrated with high accuracy for multiplex metal quantifications. After on-chip colorimetric reactions, the color did not demonstrate significant fading between 2 days to more than 4 months (SI, Preservation of color after tests).

### Detection of metals in airborne PM

**Fig. 6:**
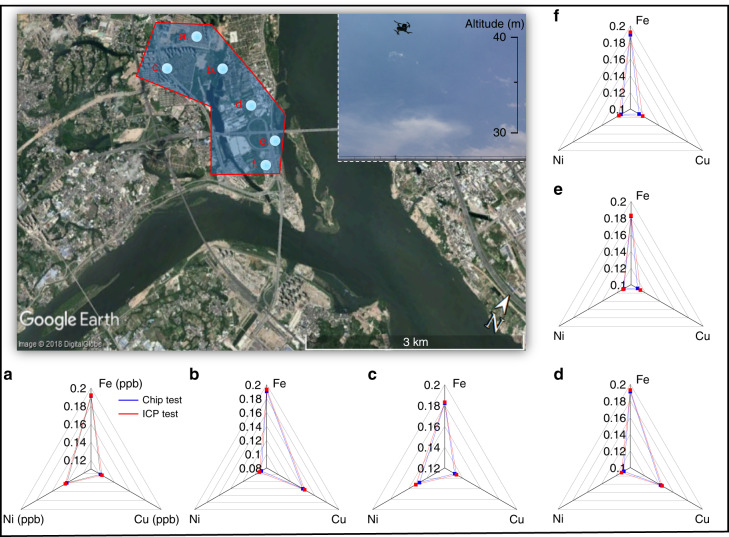
PM sample collection and quantification of on-site metal concentrations at 6 locations (~25°59′ N 119°15′ E) in Fuzhou City. **a**–**f** Quantified metal concentrations (w/w) by the portable system and ICP using the samples collected at these locations are illustrated in **a**–**f**

Finally, the system was used to quantify trace metals in airborne PM. Urban sampling sites (~25°59′ N 119°15′ E) in Fuzhou City, China, were selected, and 4-h PM samplings at 6 locations were conducted. A UAV carrying a sampler was controlled to fly and land on rooftops of 8-level buildings (altitude: ~49.2 m). After collection, the PTFE filter membranes were removed from the sampler, and half of the membrane was cut and placed in the sample pretreatment cartridge for on-site sample treatment followed by the multiplex metal assay. Notably, chelating reagents can be introduced either prior to deployment or on-site, as the expected shelf-lives of our µPADs are more than 4 weeks (SI: Shelf-life testing). As a result, the amounts of Fe, Cu, and Ni at all sampling sites were each quantified and are presented in Fig. 6. The average concentrations of these metals were determined to be 0.187, 0.126, and 0.118 ppb, respectively, (mass of metal weight/mass of air through sampler) in air. Air volume through the sampler was monitored by a digital gas flow meter (Kongxin MF5700, Nanning, China). In parallel, the other half of the PTFE filter membrane was collected and processed using standard procedures in a laboratory followed by element analysis using the ICP. The average metal concentrations were determined to be 0.189, 0.129, and 0.121 ppb for Fe, Cu, and Ni, respectively. Moreover, the same PM sample was divided and analyzed using ICP (off-site); the compositions of 5 trace metals (As, Zn, Pb, Ca, and Mn) in air PM were also detected; their concentrations (w/w) ranged from 0.03–0.11 ppb. The elements Hg, Cd, Cr, and Co were not found by ICP. Additional details are stated in SI (Local air composition analysis). By comparing the results from our portable system with those from the commercial instrument, differences of 1.0, 2.4, and 2.2% were found, thus further demonstrating the accuracy of our portable system. Additionally, the quantified average metal concentrations were lower at locations a, b, c than at locations d, e, f. This was expected, as out of the 6 locations, a, b, and c were closer to an urban area and construction sites, so that the trace metal concentrations were heavily influenced by human activity. On the other hand, d, e and f were closer to a river bank with few residential and industrial buildings. Another observation was that at similar distances, the metal concentrations were comparable between locations b and d, but relatively different between b and c. This could potentially be explained by the presence of a strong east wind on the day of the sampling. The differences in metal concentrations at such a short distance is a good example of how air pollution information can be altered at any given time and location; the results demonstrated both the effectiveness and the usefulness of our portable system in accurate, rapid quantification of trace metals concentrations in a cost-effective, low-resource setting.

## Discussion

A main disadvantage of a paper-based colorimetric assay is the heterogeneity and less desirable quality of the color distribution in the detection area, resulting in subpar detection sensitivity, and accuracy. Existing work predominately uses different bioconjugation reactions to immobilize the colorimetric ligands via carbonyl, aldehyde, or amino groups formed on the cellulose substrates. Although each of these strategies has its own advantages, they are not universally applicable, and require multiple reagents and functionalization steps to execute, which defeats the purpose of cost-efficient, simple Point-of-care testing. This paper attempts to develop a universally applicable method that not only can enhance the analytical performance of colorimetric trace metal analysis, but further holds great potential for more comprehensive applications in multidisciplinary research. Therefore, the most important hypothesis made in the paper is that the deposition of GO onto the surface of the µPads is critical in enhancing the assay detection sensitivity and accuracy.

Based on the results presented, it was demonstrated that the above hypothesis is valid. We believe this improvement can be explained by a combination of the following effects. First, as a two-dimensional carbon sheet, GO has a higher surface area (in theory, 2630 m^2^/g) than cellulose fiber-based material (e.g., paper), thus providing more assay reaction sites to increase the binding opportunities^29^ and thereby enhancing the detection sensitivity. In addition, as demonstrated by research, the transition-metal ions can be coordinated by the oxygen-rich groups on basal planes and peripheries of GO and thus be strongly absorbed^32,33^ to improve the homogeneity of assay color distribution. Consequently, this paper reveals that graphene oxide is universally applicable in enhancing the analytical performance of paper-based colorimetric detection.

Most recently, novel µPAD-based sensors have been developed that improved the detection limit of heavy-metal ions by at least 2 to 3 orders of magnitude^17,18,26,35–38^. However, the sensors were primarily used in conjunction with electrochemical detection methods, which are generally considered to be relatively complex and expensive. Furthermore, considering the concentration levels of trace metals in typical urban areas, colorimetric detection methods, although not as sensitive, are more economically efficient and practical, making the method more suitable for on-site analysis. Comparing our work with other colorimetric based sensors, we hold an advantage in integration, portability, sensitivity, and accuracy. In addition, since GO-modified μPADs do not require any form of nanomaterial functionality, this work can be considered to be the most economical. To illustrate, Table 1 below summarizes and compares the utility and analytical performances of different sensors for the quantification of heavy metals.

**Table 1:** Comparison of the utilities of analytical performances of different sensors for the quantification of heavy metals

Sensors	Methods	On-site	Metals	LOD	Ref
Paper-based optical assay plate for metal determination	CM	No	Ag	10 µM	17
µPADs coupled with solid-phase extraction for copper detection	CM	No	Cu	0.02 ng (before preconcentration	18
Radial distance based µPADs for aqueous metal detection	CM	Yes	Fe, Zn, Cu	10 ng	26
PMMA modified paper device for trace metal detection	CM	No	Co	10 µM	35
µPADs distance-based metal quantification with color screening	CM	No	Ni	0.2 mM	36
Adsorptive cathodic square-wave voltammetry for metal detection	EC	No	Co, Ni	0.05–0.25 ng	37
µPADs distance-based Cu detection using a porphyrin derivative	CM	No	Cu	10 ng	38
This work	CM	Yes	Fe, Ni, Cu	5.0 ng	

*EC* electrochemical; *CM* colorimetric

## Limitations and future work

This work has demonstrated a prototype system for on-site airborne heavy-metals quantification. The robustness of the portable system to extreme environmental parameters is limited by the UAV sampling. Specifically, UAV sampling is applicable only on sunny, partly cloudy, and cloudy days with a temperature range of 0–40 °C and a Beaufort wind-force scale of level 5 or less (below 29–38 km/h). The analysis time is still relatively long (30 min) because collected PM samples must be manually prepared before each analysis. Finally, the use of masking reagents is still necessary for multiplex testing due to a lack of specificity in all metal-ligand assays. Therefore, topics such as the design of a more specific metal-ligand complex and the development of a portable automatic sample-preparation system would be interesting to pursue in future work.

## Materials and methods

Materials and reagents were used as received from manufacturers. Whatman® filter papers (Chromatography paper, Polytetrafluoroethylene filter paper, and qualitative filter paper) were purchased from GE Healthcare (Pittsburgh, PA, U.S.). Iron chloride hexahydrate, 1,10-phenanthroline and poly (acrylic acid), acetic acid, sodium acetate anhydrous, nickel chloride hexahydrate, dimethylglyoxime, sodium fluoride, ammonium hydroxide, copper sulfate pentahydrate, dithiooxamide, sodium chloride, polyethylene glycol, sodium polyacrylate, sodium hydroxide, dimercaptosuccinic acid, triethylenetetramine, and sodium pyrophosphate were purchased from Aladdin Bio-Chem Technology (Shanghai, China). Hydrogen chloride, methanol and isopropanol were purchased from Xilong Scientific (Guangdong, China). Hydroxylamine was purchased from Sinopharm Chemical Reagent (Shanghai, China). Graphene oxide (5 mg/mL) was purchased from Tanfeng Nanotech (Suchow, Jiangsu, China). Detailed material information is listed in Table S1.

The packaged microfluidic chip consists of 3 layers. Specifically, the first layer is a cylindrical paper structure (diameter 5.0 mm, thickness 0.1 mm) kept in place by a 2.0-mm-thick PET holder with a 5.0 mm diameter through a hole in the center; together these serve as an inlet for reagents for the overall chip. The second layer is a diffusion layer. The fluidic network of this layer consists of 12 cylindrical side reservoirs (diameter 3.0 mm, thickness 0.34 mm), an inlet-connecting reservoir and microchannels bridging them. Similarly, the network is fitted in a 0.3-mm-thick PET holder so that it can guide the flow of liquids from the inlet and lead them to the third and final detection layer. Paper fluidic structures in the third layer include 12 cylindrical reactions reservoirs (diameter 3.0 mm, thickness 0.34 mm). Inserted in another 0.3-mm-thick PET holder, each of the reaction reservoirs is vertically aligned to and in contact with the 12 side reservoirs from the second layer to allow metal ions in the reagent solution to fully react with their predeposited colorimetric ligands. Finally, a 0.3-mm-thick optically transparent poly (methyl methacrylate) plate is used as an image-capture window and a ridged supporting substrate for the chip. The chip is assembled leak-free using screws and bolts. The total area of the μPAD is ~9.0 cm^2^.

The operation of the system includes three steps, on-site PM sampling and processing, on-chip colorimetric assays, and data interpretation. Starting with PM sampling, the PM sampler was reversibly mounted on the UAV with double-sided adhesive tape as shown in Fig. S1. A PTFE filter membrane with a mean pore size of 2.0 μm was chosen for air PM collecting. Sampling at designated locations was achieved by flying the sampler-mounted UAV (Mavic Pro, DJI Co., Ltd., Shenzhen, Guangdong, China). The folded UAV’s size (83 × 83 × 198 mm H × W × L) and weight (~0.74 kg) allowed easy transportation. The collected air PM samples were then digested, followed by neutralizing in the sample pretreatment cartridge. For the metals of Cu, Fe, and Ni, dithiooxamide, 1,10-phenanthroline, and dimethylglyoxime were selected as corresponding colorimetric ligands, respectively. The principle of the metal-ligand complexations is illustrated in SI (Principle of complexations). Following complete drying and device packaging, the device was inserted into a hand-held chip bin (Fig. S2). The chip bin was used to adjust the relative position of the chip and the camera (HERO6, GoPro Inc., San Mateo, CA, USA). The native image resolution of the camera was 2160 × 2160 pixels (for an image size of 30 by 30 mm). With a circular detection area with a diameter of 3.0 mm, a corresponding resolution of 200–220 pixels was obtained for each reaction reservoir. The improved image resolution represents an important improvement over a standard cellphone camera by reducing the detection bias. The chip bin was made with black carbon-fiber filament (CFPLA, 80 s Studio, Shenzhen, China) to minimize background illuminance during image capture. The dissolved PM sample was pipetted onto the chip inlet. The metal-ligand assay was then allowed to proceed to completion, after which the chip was inverted and images were taken from the backside through the poly (methyl methacrylate) window. Finally, the images were sent to a smartphone wirelessly, where they were automatically analyzed using the self-developed APP. The self-developed iOS APP uses an object’s edges tracking algorithm to automatically locate the 12 reaction reservoirs and extract values of the R, G, and B channels of each pixel in these reaction zones and convert them to a grayscale using the luminosity method.

## Supplementary information

Supporting material

GO-modified paper contact angle measurement

Paper contact angle measurement
