# Supplementary material for: Graphene oxide nanosheets coupled with paper microfluidics for enhanced on-site airborne trace metal detection
Source: Microsyst Nanoeng. 2019 Feb 11;5:4. doi: 10.1038/s41378-018-0044-z (PMC6369225; doi:10.1038/s41378-018-0044-z)
Supplement: Supplementary file 1 — Supporting material [file 41378_2018_44_MOESM1_ESM.docx]

Supporting Information for:

Graphene Oxide Nanosheets Coupled with Paper Microfluidics for Enhanced On-site Airborne Trace Metal Detection

Hao Sun^1, 2, *^, Yuan Jia^3, *^, Hui Dong^1^, Longxiang Fan^1^

1 School of Mechanical Engineering and Automation, Fuzhou University, Fuzhou, 350116, China

2 Fujian Provincial Collaborative Innovation Center of High-End Equipment Manufacturing, Fuzhou, 350001, China

3 School of Mechanical Engineering, Southeast University, Nanjing, 210096, China

**Details of experimental set-up**


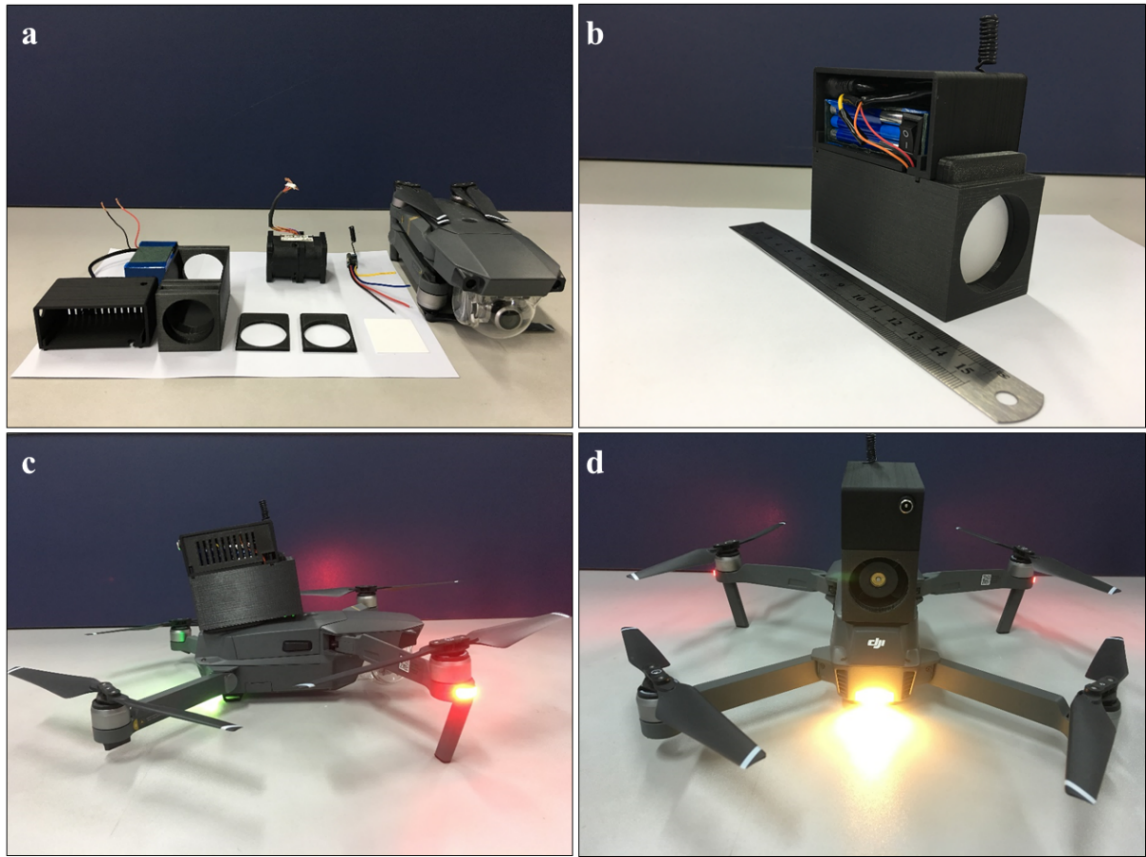


**Figure S1** UAV and self-built sampler for on-site PM collection. (**a**) Modules of PM sampler and a folded UAV. (**b**) A packaged PM sampler. (**c**) Side-view and back-view (**d**) of the PM collection platform


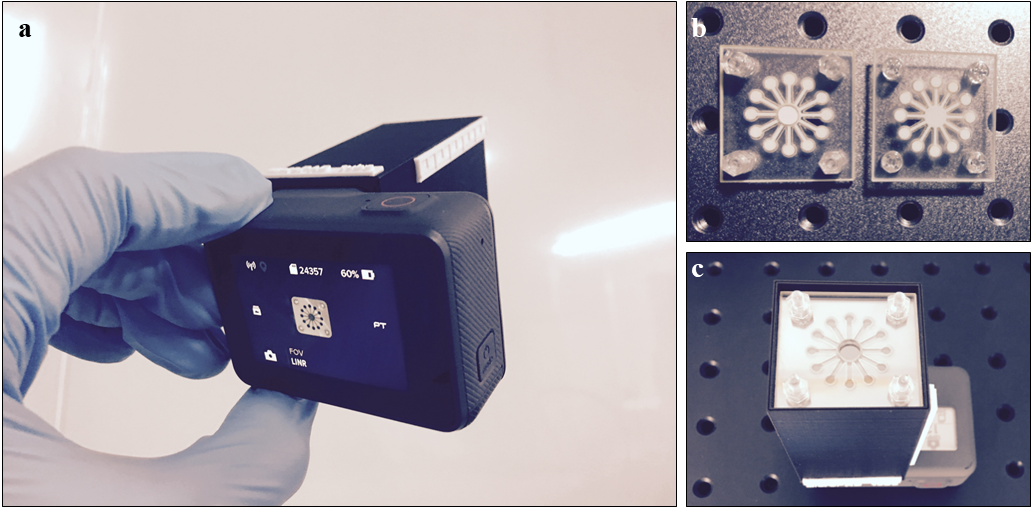


**Figure S2** Hand-held platform for portable colorimetric assay

**Sample pretreatment cartridge**

The cartridge is assembled using a 3D printed reactor, a portable polyimide-based heater and a lithium ion battery (12 V, 3600 mAh). The reactor is made with (polylactic acid) filaments and reinforced with PTFE films for optimal chemical resistance.


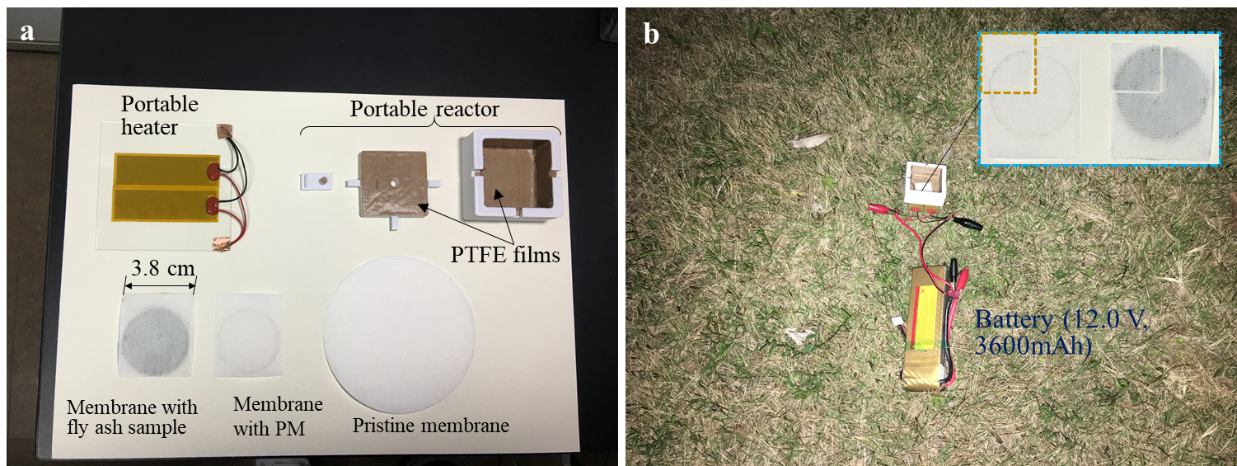


**Figure S3** (**a**) Components of field reactor kit. (**b**) An assembled field reactor kit

**Reagent preparation**

Containers were rinsed with deionization (DI) water prior to use.

**Fe detection**

Iron (III) chloride hexahydrate was used to prepare standard solution with an initial concentration of 20 mg/mL for the Fe assay. For the calibration using metal salt, the initial solution was diluted by 2~ 2000 times. 5.0 g of CH_3_COONa·3H_2_O and 2.35 mL of acetic acid was added to 10 mL DI water to acquiring a 6.3 M acetate buffer solution of pH 4.5. 40.0 mg of 1,10-phenanthroline was introduced to 5mL of the acetate buffer. 0.5 g hydroxylamine was added to the other 5 mL of the buffer. Then, 0.4 µL aliquots of hydroxylamine solution were pipetted onto the DRs and negative controls of the chip. Next, 0.3 μL of poly (acrylic acid) (0.7 mg/mL) aliquots were added to the DRs and negative controls. Finally, 0.4 μL of 1,10-phenanthroline solution was pipetted onto the targeted DR or negative control.

**Cu detection**

Copper (II) sulfate pentahydrate was used to prepare standard solution with an initial concentration of 20 mg/mL for the Cu assay. For the calibration using metal salt, the initial solution was diluted by 2~ 2000 times. Dithiooxamide (30 mM), sodium acetate buffer (pH 4.0, 20 mM) and 1%(w/w) hydroxylamine was mixed in isopropanol. Then, 0.1 µL of hydroxylamine concentration (10% w/w in DI water) was added to the targeted on-chip channel. Finally, 0.4 µL of dithiooxamide-sodium acetate-hydroxylamine buffer was added to target DR or negative control.

**Ni detection**

Nickel (II) chloride hexahydrate was used to prepare standard solution with an initial concentration of 20 mg/mL for the Ni assay. For the calibration using metal salt, the initial solution was diluted by 2~ 2000 times. The ligand buffer was prepared by adding 0.7 g of dimethylglyoxime with 100 mL of methanol. 0.1 µL of each NaF solution and acetic acid was in order pipetted onto the on-chip channel. Then, 0.4 µL of the dimethylglyoxime solution were pipetted to the DRs and negative controls. Finally, 0.4 µL of ammonium hydroxide (pH 9.5) was also pipetted onto the DR or negative control.

**Principle of the complexations**


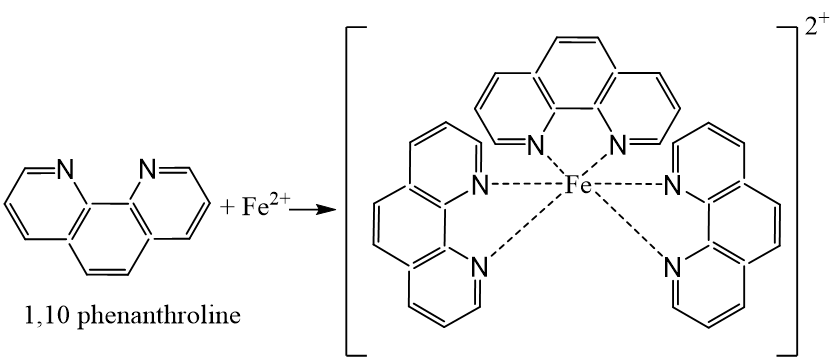


**Figure S4** Complexation of 1,10-phenanthroline and Fe


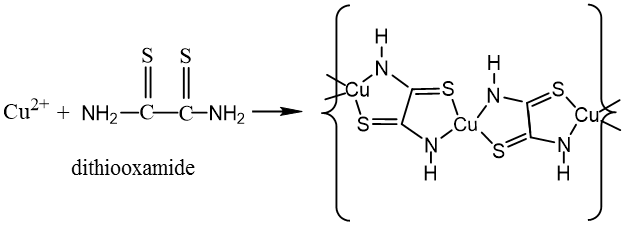


**Figure S5** Complexation of dithiooxamide and Cu


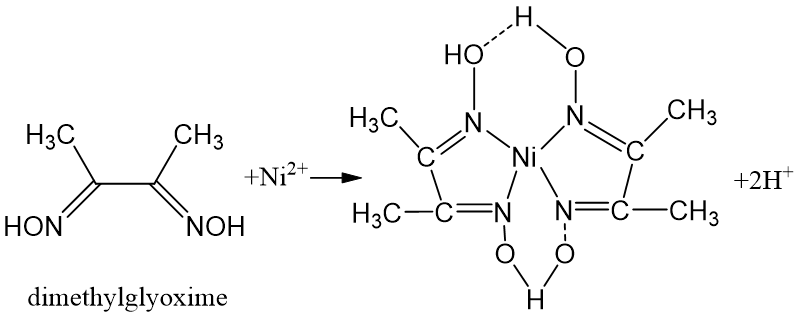


**Figure S6** Complexation of dimethylglyoxime and Ni

**Table S1** Product information of Materials

| Product | Cat. No. | Supplier |
| --- | --- | --- |
| Whatman® Chromatography Paper | 3030-861 | GE Healthcare Life Sciences |
| Whatman® PTFE filter (Φ 47 mm) | 1851-047 |  |
| Iron Chloride Hexahydrate | F102739 | AladdinBio-Chem Technology |
| 1,10-Phenanthroline | P104932 |  |
| Poly (Acrylic Acid) | P131659 |  |
| Acetic Acid | A128325 |  |
| Sodium Acetate Anhydrous | A112056 |  |
| Dimercaptosuccinic Acid | D107254 |  |
| Triethylenetetramine | T103764 |  |
| Nickel Chloride Hexahydrate | N118617 |  |
| Dimethylglyoxime | D111834 |  |
| Sodium Fluoride | S118142 |  |
| Ammonium Hydroxide | A128332 |  |
| Copper Sulfate Pentahydrate | C112401 |  |
| Dithiooxamide | D109077 |  |
| Sodium Pyrophosphate | S108847 |  |
| Sodium Chloride | C111533 |  |
| Sodium Borohydride | S108355 |  |
| Polyethylene Glycol | P103724 |  |
| Sodium Hydroxide | S128515 |  |
| Sodium Polyacrylate | S165277 |  |
| Glycine | G119904 |  |
| Isopropanol | I119459 |  |
| Dimercaptosuccinic acid | D107254 |  |
| Triethylenetetramine | T103760 |  |
| Sodium pyrophosphate | S108847 |  |
| Zinc standard | Z117295 |  |
| Mercury standard | M115418 |  |
| Calcium solution | C115397 |  |
| Arsenic solution | A110125 |  |
| Cadmium standard | C115402 |  |
| Lead standard | L115441 |  |
| Manganes standard | M105840 |  |
| Chromium solution | C105824 |  |
| Hydrogen Chloride | 3004008-01-03 | Xilong Scientific |
| Hydroxylamine | XW78034982 | Sinopharm Chemical Reagent |
| PTFE Filter Membrane (Φ 90 Mm) | Space 090 | Beijing Safelab Tech. Ltd. |

**Wettability tests of GO modified surfaces**

Wettability of microfluidic channels is able to affect the on-chip flow diffusion and mixing efficiency. Due to the highly absorbance nature of filter paper substrates, water droplets that were dispensed onto the surface of the GO modified or pristine papers were all quickly absorbed (SI-video 1, 2), making the contact angle measurements difficult and susceptible. Instead, goniometric analyses on both glass (hydrophilic) and polyethylene terephthalate (PET) sheet (hydrophobic) surfaces were made to give insights into the wettability influence of GO deposition in general. The results of contact angle (CA) tests are shown in Fig. S7 a ~ h. In the case of a glass surface (Fig. S7 a ~ d), the addition of GO for all concentrations reduced the contact angle of the water droplet, making the surface more hydrophilic. In parallel, for the case of the PET surface (Fig. S7 e ~ h), the addition of GO for all concentrations significantly reduced the droplet contact angle, changing the originally hydrophobic surface into a hydrophilic one. Notably, GO solution with a lower concentration formed a more hydrophilic surface for both cases. This can be explained by the increasing potential for GO aggregation at a higher concentration. Thus, it can be concluded that the addition of GO in a concentration of 200 μg/mL would not affect the wettability of the filter paper significantly and is most likely to make the paper more hydrophilic.


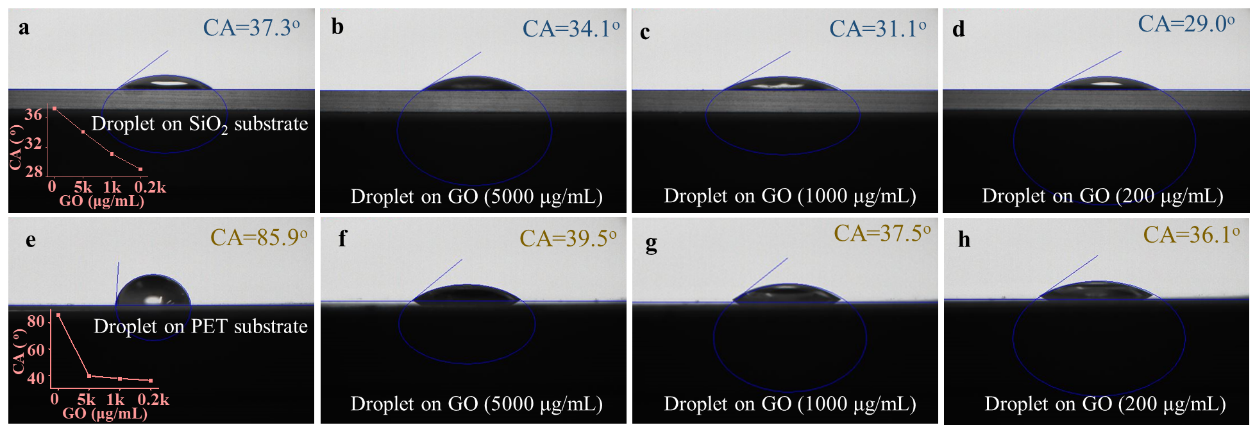


**Figure S7** Contact angle of water droplet (6 µL) on SiO_2_ glass substrate (**a** ~ **d**) and PET (**e** ~ **h**) with GO solution treatment.

### pH effect study

pH value is a critical factor for colorimetric assays. In this work, principle of complexation for Cu, Fe, and Ni detection were all standard which had been reported by previous literatures [SI-Ref. 1]. These literatures have studied the effect of pH for these complexations and given suggested pH values. Here, for further investigate the effect of pH value on the proposed tests, the suggested pH values were employed as reference medium points for each metal. Different pH values around the point were adjusted by adding NaOH or HCl solution to ligand mixtures and measured by a pH meter (PHS-3C, INESA Scientific Instrument Co., Ltd., Shanghai, China). Intensities of on-chip colorimetric reaction with different pH values are shown in Fig. S8. The results indicated that at the suggested pH value of 4.5, 4, and 9.5, the highest colorimetric intensities were found using our system for metals of Cu, Fe, and Ni, respectively. This was expected and indicated stronger bindings occurred at the reference points than other pH values.


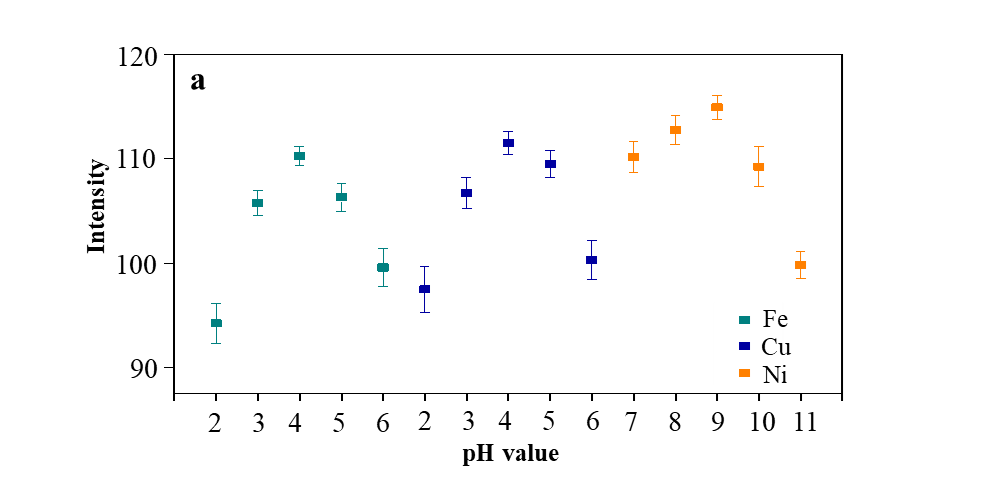


**Figure S8** Effect of pH value on colorimetric reaction

### Chelating reagent volume optimization

Dithiooxamide (30 mM), sodium acetate buffer (pH 4.0, 20 mM) and 1%(w/w) hydroxylamine were mixed in isopropanol. Then, different volume (0.2, 0.3, 0.4, 0.5 and 0.6 µL) of the mixture were pipetted onto the GO deposited detection reservoirs. After drying, Cu solution with identical concentration (2.0 µg/µL) were pipetted onto the inlet of the µPAD. Finally, the reaction signals were captured and subtracted by NTC signals as shown in Fig. S9a. With the ligand volume increasing, the intensity level also increased until the volume reaching around 0.4 µL. The phenomenon can be interpreted by the fact that the metal cations were not fully reacted with the chelator. For the volume above 0.4 µL, the signal yield kept constant indicating the reaction became saturated. Therefore, 0.4 µL was selected in the downstream tests of this work.

### GO volume optimization

Using the same concentration (200 µg/mL), different volume of GO (0.1 ~ 0.6 µL) were pipetted onto the detection reservoirs of a µPAD. Then, Cu solution with identical concentration (0.05 µg/µL) were introduced to the inlet. Finally, the reaction signals were captured and subtracted by NTC signals as shown in Fig. S9b. The results showed that the average signal intensities of Cu colorimetric assay using different GO volume were close while the standard deviations were found to be lowest at 0.3 µL. It can be explained by diffusion in paper thickness direction and instrumental error. The former may result in local concentration of GO in adjacent area of circle-shaped reservoir center inducing reaction to be less uniform on paper. The latter may be resulted from the pipette which lower limit range was 0.1 µL. Therefore, 0.3 µL GO was selected in the downstream tests of this work.


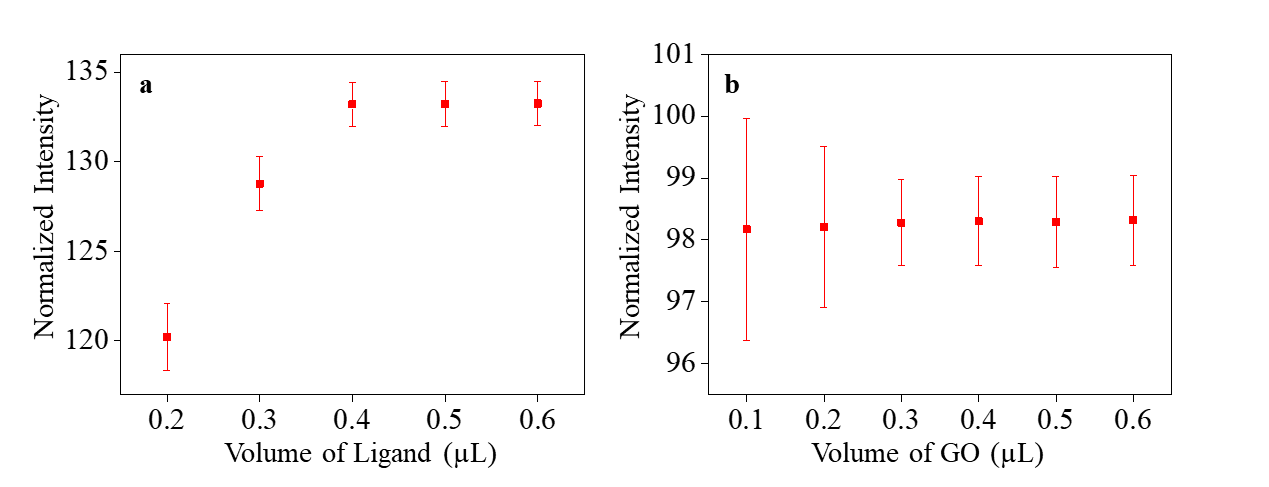


**Figure S9** (**a**) Optimization test of chelating reagent volume. Errors bars were obtained by performing 5 independent tests. (**b**) Optimization test of GO volume. Errors bars were obtained by performing 5 independent tests.

### Principle of metal inference masking

Masking reagents were used to avoid any potential interference with other metal ions. It is a process in which a substance, without physical separation of it or its reaction products, is so transformed that certain of its reactions are prevented. Optimum conditions coupled with suitable masking reagents can offer highly selective reactions. In general, a chelator (or chelating agent) is used for the binding of metal ions to a macromolecule and forming a ring with the metal atom. For different targeted metal ions, we used specific combinations of chelators to mask other potential ions in multiplex testing. Specific chelator combination for Fe, Cu and Ni is shown in Figure S10 and the complexation principle of the three metals are shown in Figure S11 ~ S13. The pH values of masking reagents were adjusted with NaOH and HCl without additional salts.


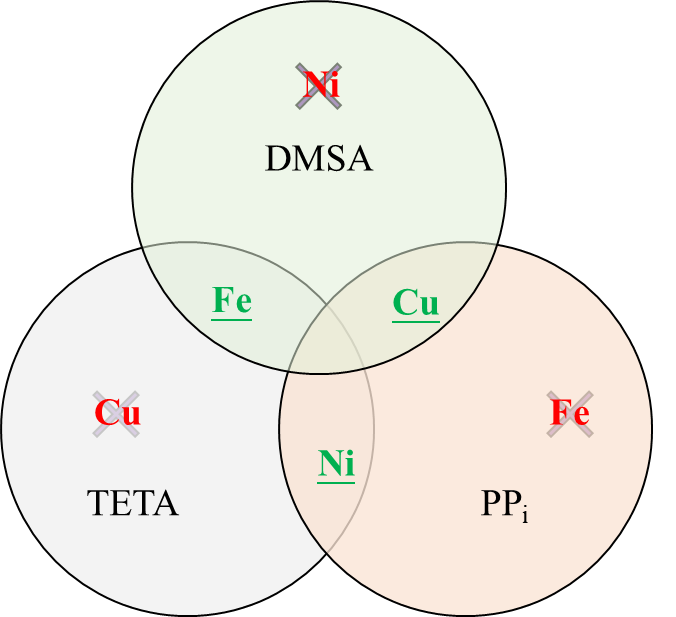


**Figure S10** Schematic of the masking reagent combination for Fe, Cu and Ni detection. (DMSA: Dimercaptosuccinic acid; TETA: Triethylenetetramine; PP_i_: pyrophosphate)


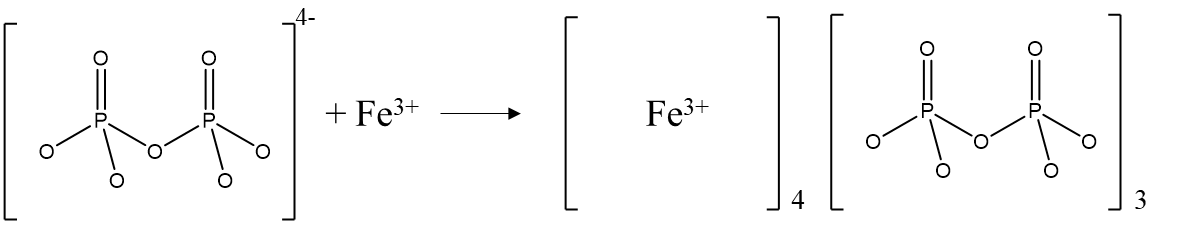


**Figure S11** Complexation of PP_i_ and Fe (pH value=10.0±0.2, SI-Ref. [2])


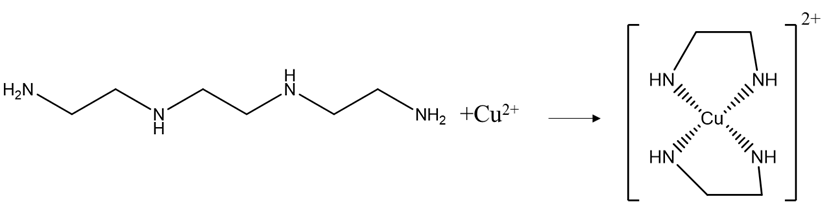


**Figure S12** Complexation of TETA and Cu (pH value=10.0±0.2, SI-Ref. [3])


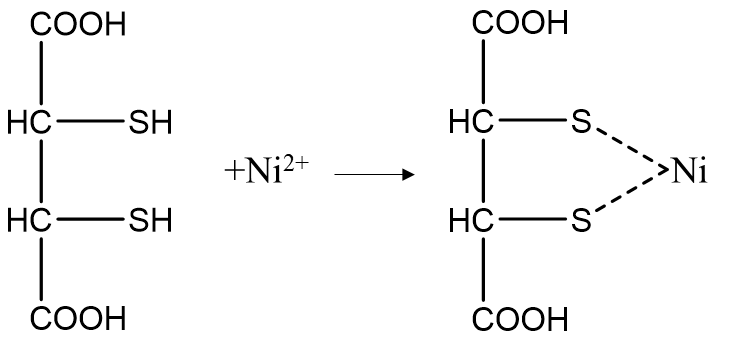


**Figure S13** Complexation of DMSA and Ni (pH value=9.5±0.2, SI-Ref. [4])

**Metal interference study**

Masking reagents preparation for the on-site Fe, Cu and Ni detection followed a recently published protocol^34^. In details, aqueous solutions of dimercaptosuccinic acid (0.18 g/10 ml) and triethylenetetramine (0.15 g/10 ml), dimercaptosuccinic acid (0.18 g/10 ml) and pyrophosphate (0.33 g/10 ml), pyrophosphate (0.33 g/10 ml) and triethylenetetramine (0.15 g/10 ml) with an adjusted pH value of 10 were precoated onto designated chip DRs for the detection of Fe, Cu and Ni, respectively (Table S2).

**Table S2** Masking Reagent (per 10 mL)

| Metal | Masking Reagents | Mass (g) |
| --- | --- | --- |
| Fe | Dimercaptosuccinic acid | 0.18 |
|  | Triethylenetetramine | 0.15 |
| Cu | Dimercaptosuccinic acid | 0.18 |
|  | Pyrophosphate | 0.33 |
| Ni | Pyrophosphate | 0.33 |
|  | Triethylenetetramine | 0.15 |

Interfering metals with a 10-fold concentration increase and intra-assay control were introduced to dedicated reservoirs (Fig. S14a and 14b). For each target metal, same reagents in identical order, only without the interfering metals, were introduced to a second µPAD to serve as a comparison. Finally, only GO solution and chelating reagents were introduced to a third µPAD to serve as inter-assay NTC templates. After the completion of the colorimetric assays, one example group of on-chip assay images was captured as shown in Fig. S14c.


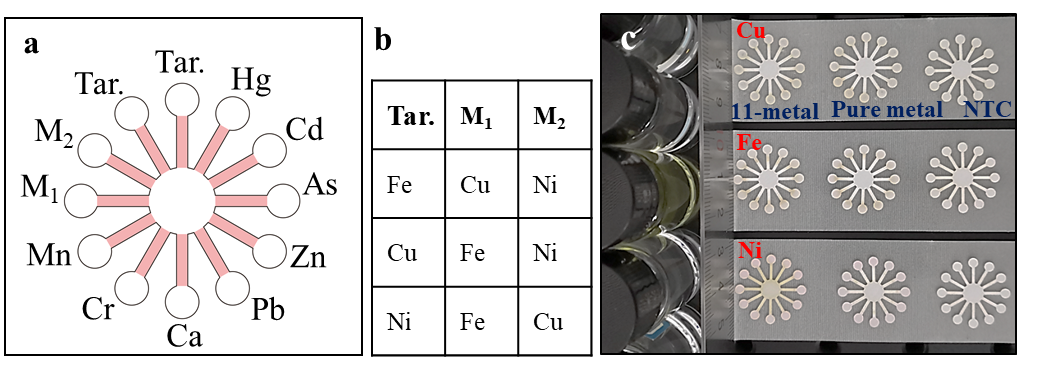


**Figure S14** (**a**) The layout of µPADs during interference tests. (**b**) Cross-reference table for different metal targets. (**c**) Colorimetric assay images of target metals crossed referenced with 10 interfering metal ions.

**Calibration using metal salt solutions**

The portable system was first calibrated using metal salt solutions. Cu, Fe, and Ni metals in salt form were directly dissolved in DI water and diluted into predetermined concentrations. Details of reagent preparation and interference masking treatment for the samples are described in Table S2.

The calibration experiments involved two steps: the first step was the overall inter negative control experiment. It started with pipetting 0.3 µL of well-dispersed GO solution onto the center of the 12 detection reservoirs. In parallel, all 12 side reservoirs of the second layer were introduced with 0.3 µL of perspective masking reagents based on metal sample constituents (Table S2). Also, 0.4 µL of the corresponding ligand solution was pipetted onto 11 of the 12 reaction reservoirs with the last reservoir serving as the intra negative control. The chips were packaged and allowed to dry at room temperature, and 5.0 μL of pure buffer was then pipetted onto the chip inlet. The Wi-Fi camera was used to capture the images of reaction reservoirs through the backside PMMA window, and the images were then processed by the APP. The result of this experiment, the average background grey intensity value, was then recorded and used as the overall experimental baseline.

The second step was the metal-ligand assay experiments. The process was identical to that of the control experiment except 5.0 μL of each metal salt solutions was introduced to the inlet instead. To determine the average gray intensity value for each metal assay as a function of metal concentrations, baseline grey values obtained from both inter/intra negative control were subtracted.

To minimize the detection bias caused by chip flow resistance, the maximum and minimum intensity values all reaction reservoirs readouts were excluded prior to grey intensity calculations.

**On-site trace metals multiplex quantification**

The process of on-site trace metals quantification was similar to the calibration experiments. However, the trace metals quantifications used a multiplex detection format so that the masking reagents were precoated to 3 of the 12 reaction DRs, respectively, and the remaining 3 reaction reservoirs served as the intra negative control. In addition, for on-site air PM processing, samples collected in air were first digested using 100 μL of nitric acid (80%), and the solution was then condensed by heating to 60 ^o^C for 20 minutes using the on-chip heater to fully dissolve the PM. The condensed solution was allowed to cool and was neutralized by adding 160 μL of sodium bicarbonate (0.5 M) in the cartridge. 5 µL of the resulting solution were then withdrawn and disposed at the chip inlet, and the metal-ligand assays were allowed to proceed to completion. The Wi-Fi camera was again used to capture the images followed by data processing using the APP.

### Raw images of on-chip reaction


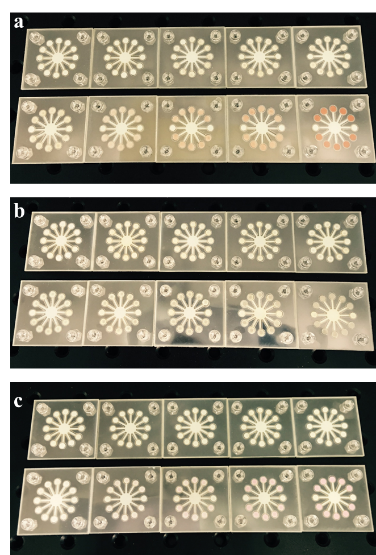


**Figure S15** Raw images of on-chip reaction.

### Statistical comparison of on-chip and ICP testing results

Tests were performed for Fe, Cu, and Ni with a sample size of 10 (7 additional samples). Solution mixture that included Cu, Fe, and Ni ion with unknown concentrations were tested by on-chip approach and ICP. Then, using the software IBM SPSS Statistics (version 24), the means of the two data sets were compared by methods of independent-samples T test (t-test) and one-way ANOVA (F-test). The Sig. values of t-test and F-test based on different metal tests were calculated and shown in Table S3 (Fe), Table S4 (Cu), and Table S5 (Ni). The values were all above the threshold chosen (0.05 level) for statistical significance.

**Table S3** t-test and F-test for comparing the means of on-chip and ICP based Fe quantification

| Fe Test# | X_1_ (ppm, On-site using the portable system ) | X_2_ (ppm, Off-site by ICP) |
| --- | --- | --- |
| #1 | 9.52 | 8.89 |
| #2 | 8.06 | 8.19 |
| #3 | 10.27 | 10.29 |
| #4 | 10.29 | 10.09 |
| #5 | 8.29 | 10.15 |
| #6 | 10.58 | 9.06 |
| #7 | 9.99 | 9.69 |
| #8 | 9.08 | 9.28 |
| #9 | 8.59 | 10.57 |
| #10 | 10.68 | 10.23 |
| $\overline{X}$ | 9.535 | 9.644 |
| F-values (F-test) | 0.078 | |
| Sig. (F-test) | 0.784 | |
| T-values | 0.279 | |
| Sig. (T-test) | 0.294 | |
| Sig.(2-tailed) | 0.784 | |

**Table S4** t-test and F-test for comparing the means of on-chip and ICP Cu quantification

| Cu Test# | X_1_ (ppm, On-site using the portable system) | X_2_ (ppm, Off-site by ICP) |
| --- | --- | --- |
| 1 | 10.59 | 11.97 |
| 2 | 11.06 | 12.16 |
| 3 | 11.97 | 12.15 |
| 4 | 12.32 | 12.30 |
| 5 | 12.18 | 11.58 |
| 6 | 11.37 | 11.26 |
| 7 | 12.24 | 10.69 |
| 8 | 10.96 | 10.71 |
| 9 | 10.68 | 11.18 |
| 10 | 10.42 | 11.51 |
| $\overline{X}$ | 11.379 | 11.551 |
| F-values (F-test) | 0.33 | |
| Sig. (F-test) | 0.573 | |
| T-values | 0.575 | |
| Sig. (T-test) | 0.262 | |
| Sig.(2-tailed) | 0.573 | |

**Table S5** t-test and F-test for comparing the means of on-chip and ICP Ni quantification

| Ni Test# | X_1_ (ppm, On-site using the portable system) | X_2_ (ppm, Off-site by ICP-OES) |
| --- | --- | --- |
| 1 | 11.69 | 11.61 |
| 2 | 11.54 | 10.41 |
| 3 | 10.37 | 10.83 |
| 4 | 11.96 | 11.87 |
| 5 | 11.89 | 11.84 |
| 6 | 11.01 | 11.46 |
| 7 | 10.29 | 11.01 |
| 8 | 10.48 | 10.82 |
| 9 | 10.33 | 10.97 |
| 10 | 10.58 | 11.24 |
| $\overline{X}$ | 11.014 | 11.204 |
| F-values (F-test) | 0.508 | |
| Sig. (F-test) | 0.485 | |
| T-values | 0.713 | |
| Sig. (T-test) | 0.084 | |
| Sig.(2-tailed) | 0.485 | |

### Local air composition analysis

After PM collection, same batch of sample was divided and analyzed using both the portable system (on-site) and the ICP (off-site). The airborne heavy metal composition at the sampling sites were then analyzed by ICP and given in a table format below.

Table S6 Metals in the PM sample detected by ICP-MS (ppb)

|  | Fe | Cu | Ni | Hg | Cd | As | Zn | Pb | Ca | Cr | Co | Mn |
| --- | --- | --- | --- | --- | --- | --- | --- | --- | --- | --- | --- | --- |
| Mass ratio  (metal/air) | 0.19 | 0.13 | 0.12 | N/A | N/A | 0.11 | 0.03 | 0.04 | 0.05 | N/A | N/A | 0.06 |

**Preservation of color after tests**

In an open environment, the fading will not occur for at least a day as the top and bottom surface of our 3D-μPADs were sealed with PET film, only exposing the inlet (diameter of 5.0 mm) to air. In addition, after transferring the μPADs to a low humidity environment (e.g. a thermostatic environmental chamber), the color of Fe remains consistent for a period of at least 4 months. For Cu, the color fading was observed after 2 weeks and the intensity was reduced to 65% after 4 months. For Ni, the color fading was observed after 3 days and the intensity was reduced to about 0% after 4 months. The chip presented in main text Fig. 5h was captured again after about 4 months as shown in Fig. S16.


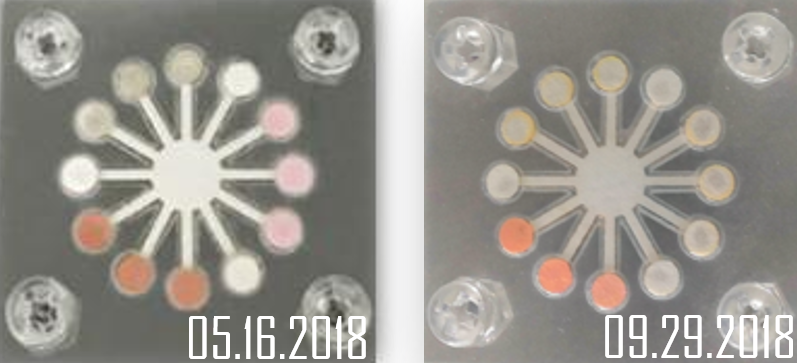


**Figure S16** Changing in µPAD color intensity after a period of 4 months.

**Shelf-life testing**

**Shelf-life testing**

To evaluate the shelf-life of the device, the chelating reagents of Fe, Cu and Ni were pipetted onto chips. The devices were packaged and tightly sealed by multilayers of PET and enclosed by a light-proof bin. At room temperature (25 °C) and general humidity (50 %), the devices were stored for various time duration (1 ~ 4 weeks). Then, at different time point, the devices were exposed to Fe, Cu and Ni solutions (0.05 µg/µL). The assay intensity changes under different storage time for all metals were shown in Fig. S17. Until the maximum of the storage period (4 weeks), the devices showed no distinguishable variation in assay intensities. Therefore, the presented approach and device is expected to be stable at least for 4 weeks by ordinary storage.


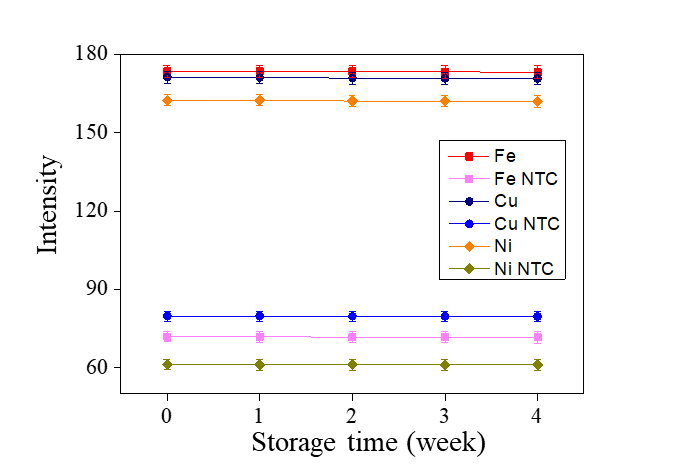


**Figure S17** Shelf-life testing

### SI-Reference

[1] Mentele, M. M., Cunningham, J., Koehler, K, Volckens, J., Henry, C. S. Microfluidic paper-based analytical device for particulate metals. *Anal. Chem.* 2012; **84**, 4474-4480.

[2] Chavada, V. D., Bhatt, N. M., Sanyal, M., Shrivastav, P. S. Pyrophosphate functionalized silver nanoparticles for colorimetric determination of deferiprone via competitive binding to Fe (III). *Microchim. Acta* 2017; **184**, 4203-4208.

[3] Henriet, T., Gana, I., Ghaddar, C., Barrio, M., Cartigny, Y., Yagoubi, N., Rietveld, I. B. Solid state stability and solubility of triethylenetetramine dihydrochloride. *Int. J. Pharmaceut.* 2016; **511**, 312-321.

[4] Mekada, T., Yamaguchi, K., & Ueno, K. Use of masking agents in chelatometric titrations—IV: 1 Dimercaptosuccinic acid. *Talanta*, 1964; **11**, 1461-1464.
